# Coccygeal Nerve Blockade vs. Impar Ganglion Blockade in Coccydynia: A Randomised Clinical Trial

**DOI:** 10.7759/cureus.69118

**Published:** 2024-09-10

**Authors:** Gevher Rabia Genc Perdecioglu, Gokhan Yildiz

**Affiliations:** 1 Department of Algology, Ankara Etlik City Hospital, Ankara, TUR

**Keywords:** coccydynia treatment, coccyx pain, fluoroscopy intervention, nerve block, peripheral nerve ultrasonography

## Abstract

Background and objective

Coccydynia is a condition that causes pain around the coccyx, severely limiting functionality. Interventional treatment options are available for cases that do not respond to conservative treatment. Coccygeal nerve block (CnB) is a novel method for treating coccydynia. This study compared the efficacy of CnB and ganglion impar block (GiB) and aimed to evaluate the efficacy of CnB in treating coccydynia.

Methods

The 12-week results of 56 patients were analysed. One group underwent ultrasound (US)-guided CnB, while the other group underwent fluoroscopy (FL)-guided GiB. Pain intensity was assessed using the numerical rating scale (NRS), and functionality was assessed using the PARIS Coccydynia Functionality Questionnaire. Evaluations were conducted before treatment, as well as four and 12 weeks after treatment.

Results

The baseline median NRS score was eight in both groups, while at week 12, it was three in the CnB group and 2.5 in the GiB group. The median PARIS score at baseline was seven in the CnB group and 6.5 in the GiB group, while at week 12, it was four and three, respectively. At week 12, compared to baseline, both the CNB and GiB groups showed statistically significant improvements in NRS and PARIS scores (p<0.001). When comparing the two groups, no significant difference was observed in the NRS and PARIS scores before and four and 12 weeks after treatment. No serious adverse events were observed in any patient.

Conclusions

Coccydynia is sometimes refractory to treatment. In our study, we found that CnB, a method recently used in the treatment of coccydynia, was as effective as GiB, which has been used for a long time, and we found no evidence of superiority. The lack of radiation exposure due to its US-guided application, the superficial course of the coccygeal nerves and the low depth of needle penetration make it easier to perform. These advantages suggest that it will be a preferable method in the treatment of coccydynia.

## Introduction

Coccydynia is a condition characterised by pain around the coccyx, which can be caused by musculoskeletal conditions, infection, or malignancy. The etiology is often attributed to trauma or childbirth. Risk factors include female gender, obesity, rapid weight loss, variations in coccygeal morphology, and coccygeal hypermobility. It is more prevalent in middle-aged women [1,2].

Increased pain with sitting is a typical clinical presentation for coccydynia. Pain can be worsened by getting up from a sitting position, prolonged standing, the premenstrual period, sexual intercourse, and bowel movements [3].

Diagnosis of coccydynia is made using clinical and imaging techniques. Magnetic resonance imaging (MRI) can visualise the sacrococcygeal joints and the distal coccyx bone and rule out secondary pathologies such as infection and tumours in this region. Dynamic MRI may also be used to diagnose coccygeal movement [4,5]. In patients refractory to conservative management, interventional procedures such as steroid injections, caudal epidural injection, ganglion impar block (GiB), and spinal cord stimulation are available prior to coccygectomy [6].

The impar ganglion, located anterior to the coccyx, is the end point of the sympathetic chain and is responsible for nociception and sympathetic innervation of the perineal region [7]. It is not yet clear how sympathetic blockade improves somatic pain. Local anaesthetics, steroids or combinations, and also radiofrequency, pulsed or conventional, are applied to the impar ganglion and are successful.

The coccygeal nerve is composed of the coccygeal plexus and is responsible for receiving sensation from the coccyx region. Upon exiting the sacral hiatus, the coccygeal nerves travel inferiorly, passing medial to the coccygeal cornu and laterally under the transverse process of the first coccygeal vertebra. The nerve follows a superficial course at this level [8].

There was limited literature data on coccygeal nerve block (CnB) [9,10]. Two new articles on coccygeal nerve intervention for coccyx pain have recently been published. The articles report successful results using an ultrasound (US)-guided nerve identification method at the level of the coccygeal cornu [11-19]. According to Sencan, sympathetic blockade results in inhibition of the neuropathic component of coccydynia. In addition, steroids prolong the blockade effect [20].

The primary aim of this study was to compare the efficacy of CnB, a new method in the treatment of coccidynia, with GiB, an established method. Secondary aims were to evaluate the effect of the interventions on the functionality score and to determine the adverse events associated with the interventions.

## Materials and methods

This study was conducted as a randomised, controlled, and prospective trial. Ethics committee approval was obtained from the local hospital, and the Clinical Trial submission system was registered (Clinical Trial Number NCT06242587). This study was conducted in the single-center pain clinic of a tertiary care hospital. Informed consent was obtained from all participants. We used a computer-assisted randomisation program to categorise patients into groups. The other researcher as an independent statistician, unaffiliated with the enrollment of participants, produced the random allocation sequence. This sequence was retrieved through a secure system online. To maintain the integrity of allocation concealment, treatment assignments were enclosed in sealed, nontransparent envelopes. The interventions were performed by a physician with at least five years of experience in US and fluoroscopy (FL). The researcher who collected and analyzed the patients' pain and functionality scores did not know which patient received which intervention. 

Participants

The inclusion criteria included age between 18 and 70 years, coccydynia >3 months, unresponsive to conservative treatments (such as medication, sitting cushion, physiotherapy), and confirmation of the diagnosis of coccydynia by an MRI.

The exclusion criteria included those with a secondary pathology detected by an MRI (malignancy, infection), pregnancy, presence of additional rheumatological diseases, L4-L5-S1 discopathy, diabetes mellitus, interventional procedures or for coccydynia in the last six months, and previous coccyx surgery.

Interventions

All procedures were performed without sedation, under local anaesthesia, and in sterile conditions. Patients were monitored according to the American Society of Anesthesiologists (ASA) standards. A mean arterial pressure (MAP) below 65 mmHg for at least one minute or a drop of more than 20%-30% of MAP during the procedures was defined as hypotension and treated with intravenous crystalloids and vasopressors (usually ephedrine). 

CnB

**Figure 1:**
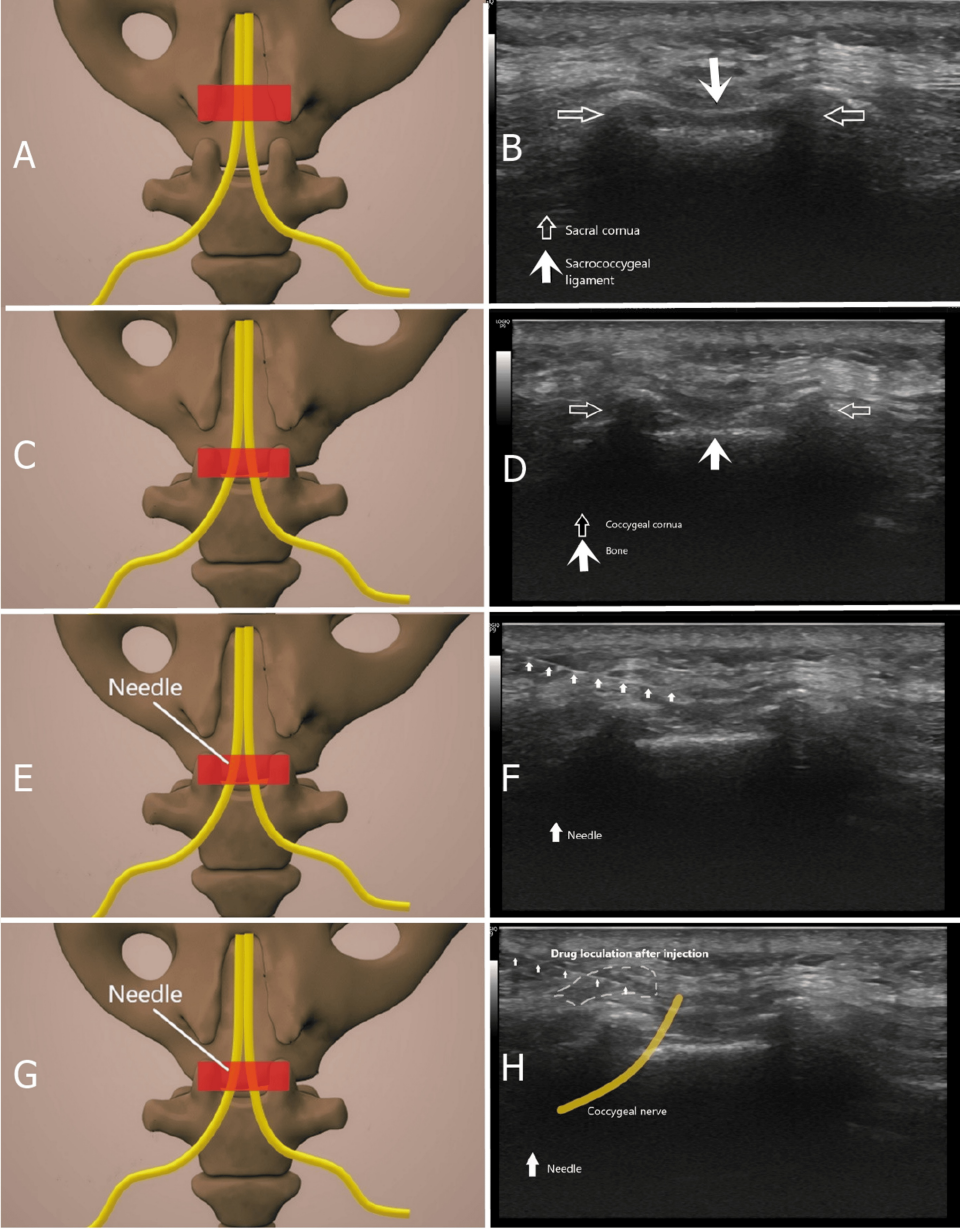
Coccygeal nerve blockade A shows the ultrasound probe (red rectangle) positioned over the sacral cornu, while B shows the ultrasound image of the sacral cornu and the sacrococcygeal ligament at this probe position. C shows the ultrasound probe advanced caudally toward the coccygeal cornu, while D shows the ultrasound image of the coccygeal cornu at this probe position. E shows the position of the needle insertion relative to the ultrasound probe, while F shows the ultrasound image of the needle on the coccygeal horn. G shows the position of the needle insertion relative to the ultrasound probe, while H shows the possible position of the coccygeal nerve on the ultrasound image and the distribution of the administered drug. Red rectangle: Ultrasound probe

Under US guidance, a CnB was performed in the intervention room. The patient was positioned prone and covered with a sterile drape. Using a 5-12 MHz linear US probe (LOGIQ P9, GE Ultrasound, Seongnam-si, Gyeonggi-do, Korea), the sacral cornuas and sacrococcygeal ligament were visualised. The transducer was then advanced 2-3 mm caudally to visualise the coccygeal cornuas. The skin and subcutaneous tissue were anaesthetised with 1% lidocaine using a 27-gauge needle. Using the in-plane technique, a 22-gauge spinal needle was inserted superomedial to the coccygeal cornu. Negative aspiration was confirmed before injecting 1 mL (4 mg) of dexamethasone (to prolong the duration of pain relief and for anti-inflammatory activity) and 1 mL (5 mg) of bupivacaine 0.5% for each nerve (8 mg dexamethasone and 10 mg bupivacaine total for each patient). Patients were monitored for possible complications for two hours after the procedure. Figure 1 shows the probe placement and US images for coccygeal nerve blockade.

GiB

**Figure 2:**
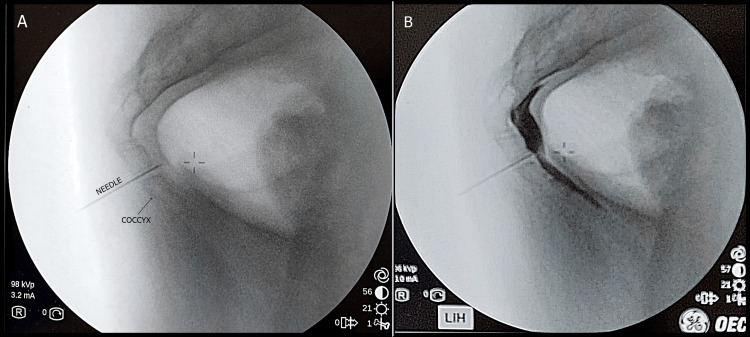
Ganglion impar blockade A shows the needle passing through the intervertebral space to the anterior side of the coccyx for ganglion impar blockade, and B shows the appropriate contrast agent spread

The GiB was performed in the operating room under FL guidance using a transcoccygeal approach. The patient was placed in the prone position, and the skin was sterilised and draped. The level of the procedure was determined using a lateral C-arm FL view. The skin and subcutaneous tissue were anaesthetised with 1% lidocaine using a 27-gauge needle. Once the target area was reached with a 22-gauge spinal needle, 2 cc of contrast medium (iohexol, 300 mg iodine/ml, GE Healthcare) was administered. Contrast agent was observed as a reverse coma sign, which is typical of an GiB. After negative aspiration, a total of 2 mL (8 mg) of dexamethasone, 2 mL (10 mg) of 0.5% bupivacaine, and 2 mL of saline were injected. Patients were monitored for side effects for two hours. Figure 2 shows the needle placement and fluoroscopic images for GiB.

Outcome measurements

The primary outcome of this study was to evaluate and compare the changes in pain intensity between CnB and GiB using the numerical rating scale (NRS) score. The NRS scale was used to assess pain intensity. Patients were shown a template with numbers ranging from 0 (no pain) to 10 (most severe pain) and were asked to mark the intensity of their pain. The NRS scale was marked three times: before the procedure, four weeks after the procedure, and 12 weeks after the procedure.

One of the secondary outcomes of this study was to analyse changes in the functional status of the patients using the PARIS functional questionnaire. This questionnaire includes self-administered questions about pain during various activities and assesses the relationship between pain and physical activity on a scale of 0 to 10. The questionnaire was completed by patients at the initial assessment and 12 weeks after the procedure. The other secondary outcome was the detection of adverse events related to the interventions.

Statistical analyses and sample size

All analyses were performed with the jamovi project version 2.3 (Released 2022; Jamovi, Computer Software, Sydney, Australia). The results of the study are expressed as frequencies and percentages. Normality analysis was performed using the Shapiro-Wilk test, skewness-curvature, and histograms. Normally distributed variables are presented as mean and standard deviation (SD). Categorical variables were compared using the chi-squared test. Numerical dependent variables were compared between groups using independent samples t-test and Mann-Whitney U test. Repeated measures were analysed using Friedman and Wilcoxon tests. Changes over time were compared using the Bonferroni correction. p < 0.05 was considered statistically significant.

The sample size was based on primary outcomes and calculations performed with the G*Power version 3.1.9.4 software program (Released 2019; Heinrich-Heine-Universität Düsseldorf, Germany) by taking an effect size 0.692, α = 0.05, power (1-β) = 0.90. A total of 37 individuals were reached for each group. Sencan et al.'s third-week NRS scores (mean and SD values) were obtained for this analysis [21].

## Results

**Figure 3:**
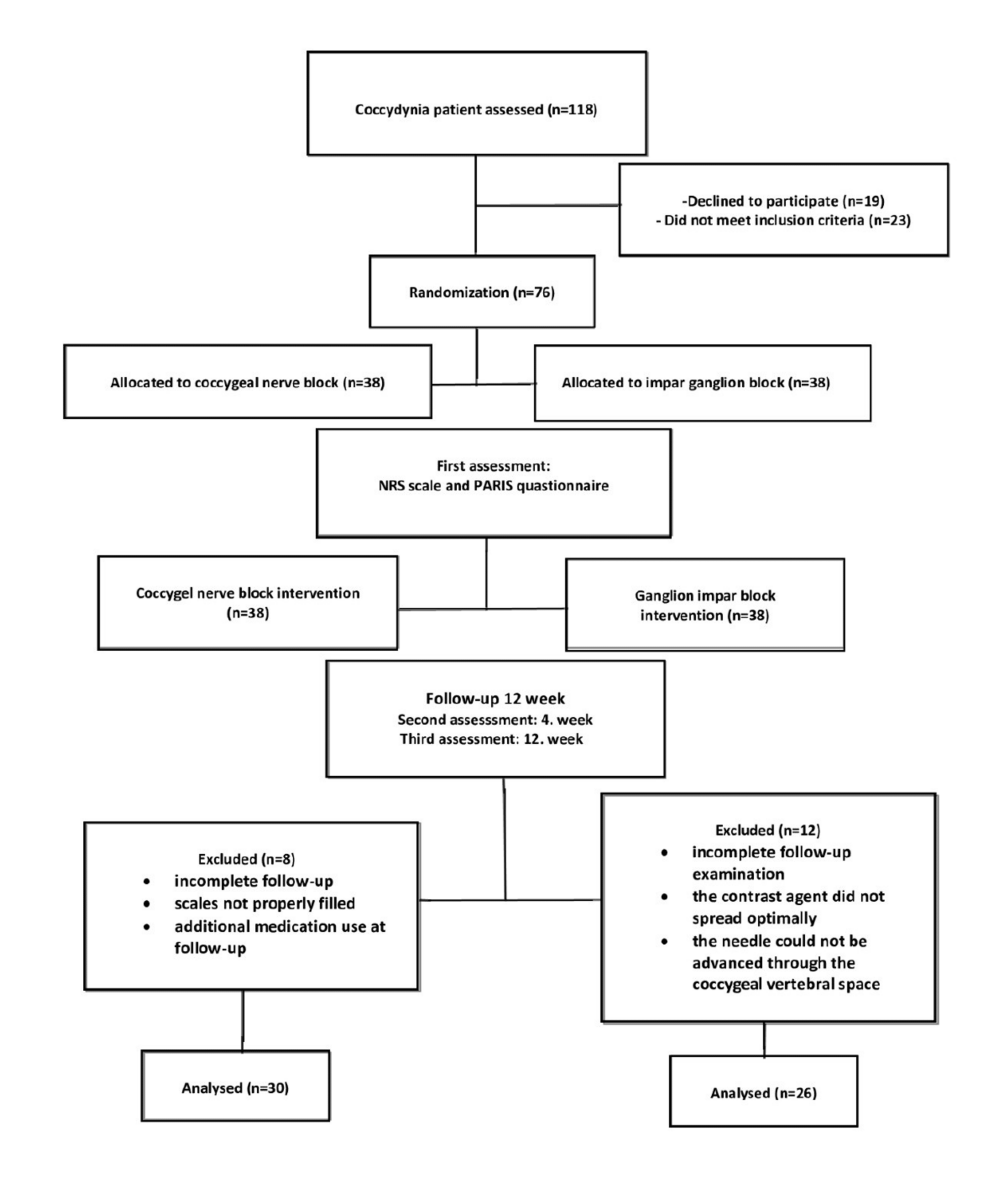
Flowchart diagram

In total, 118 patients with coccydynia were screened for eligibility. A total of 19 patients refused to participate in the study and 23 patients did not meet the inclusion criteria. A total of 76 patients were randomised into CnB and GiB groups, with 38 patients in each group, and interventions were performed. After exclusion during the follow-up period, the study was completed with 30 patients in the CnB group and 26 in the GiB group. A patient flowchart is shown in Figure 3. 

There was no difference in age, sex, and aetiology between the two groups (Table 1). In the NRS analyses between groups, no difference was found at baseline. There were also no differences between the groups at four and 12 weeks. As a result, no superiority was found between CnB and GiB on pain intensity (Table 1). Changes in NRS score were analysed within groups. The decrease in NRS score at 12 weeks was statistically significant in both groups (p < 0.001 for both groups). The change between baseline to four weeks and baseline to 12 weeks was statistically significant in both groups, whereas the change between the four- and 12-week measurements was not significant (Tables 1-2).

**Table 1:** Demographic and clinical characteristics a: Independent sample T-test; b: Chi-square test; c: Mann-Whitney U test Group CnB: Group coccygeal nerve block; Group GiB: Group ganglion impar block; NRS: numerical rating scale; PARIS: PARIS Coccydynia Functionality Questionnaire; F: female; M: male; I: idiopathic; T: traumatic

	Group CnB (n = 30)		Group GiB (n = 26)		
Characteristics	mean ± std.dev	med (min-max)	mean ± std.dev	med (min-max)	p
Age	42.40 ± 8.52	44 (26-57)	41.27 ± 13.59	13,59 (20-70)	0,479^a^
Sex (F/M)	21 (70%)/9 (30%)		21 (80.8%)/5 (19,2%)		0.353^b^
Aetiology (I/T)	17 (56.7%)/13 (53.8%)		12 (46.2%)/14 (53.8%)		0.605^b^
Outcomes					
NRS basal	7.70 ± 1.14	8 (6-9)	8.12 ± 1.03	8(6-9)	0.163^c^
NRS week 4	3.90 ± 2.32	4 (1-8)	3.23 ± 2.38	2(1-9)	0.293^c^
NRS week 12	4.13 ± 2.24	3 (1-8)	3.38 ± 2.43	2.5(1-9)	0.236^c^
PARIS basal	6.97 ± 1.49	7 (4-9)	6.65 ± 1.23	6.5(5-9)	0.401^c^
PARIS week 12	4.07 ± 1.46	4 (2-6)	3.58 ± 1.69	3(2-8)	0.245^c^

**Table 2:** Temporal variation of scale scores a: Friedman Test; b: Wilcoxon Test; *: statistically significant difference compared to baseline; Group CnB: group coccygeal nerve block; Group GiB: group ganglion impar block; NRS: numerical rating scale; PARIS: PARIS Coccydynia Functionality Questionnaire

		NRS		PARIS	
		Median (min-max)	p	Median (min-max)	p-value
Group CnB	Basal	8 (6-9)	<0.001a	7(4-9)	<0.001b
	Week 4*	4 (1-8)			
	Week 12*	3 (1-8)		4 (2-6)	
Group GiB	Basal	8 (6-9)	<0.001a	6.5 (5-9)	<0.001b
	Week 4*	2 (1-9)			
	Week 12*	2.5 (1-9)		3 (2-8)	

The PARIS Coccydynia Functionality Questionnaire scores between the groups were analysed at baseline and week 12. There were no differences between baseline and week 12 scores between groups. When analysed within groups, the decrease in PARIS scores observed at week 12 was statistically significant (p < 0.001 for both groups) (Tables 1-2).

During the treatment, two patients in Group CnB experienced numbness at the injection site, and one patient experienced hyperalgesia. In Group GiB, one patient had the needle tip advanced a little too far, but it did not cause rectal perforation. Antibiotic treatment was started prophylactically. Two patients experienced hypotension, and one patient had a transient increase in pain. Hypotension was mild and appropriately treated with crystalloid infusion without complications. These mild adverse events resolved spontaneously within 18 hours in both groups, with the longest lasting without the need for treatment. No major complications were observed with either intervention.

## Discussion

This is the first randomised controlled trial to evaluate the efficacy of US-guided CnB at the level of the coccygeal cornuas in the treatment of coccydynia. After 12 weeks of follow-up, both GiB and CnB were found to have similar efficacy in reducing pain and improving function in patients with chronic coccydynia.

Pericococcygeal injection, caudal epidural injection, and GiB are methods in persistent coccydynia. Injection content varies as a local anaesthetic, steroid, dextrose, and combinations [22]. There is currently no consensus on the most effective method, injection content, or injection sites.

Pericoccygeal injections can be considered a first-line injection method because they are minimally invasive and can be performed with a blind technique. This technique can be performed using US guidance or blind technique, but FL is rarely used. The injection site is typically located at the sacrococcygeal junction or most painful points. Conflicting data exists regarding its effectiveness [23-25].

Caudal epidural injection is another frequently used method. It can be performed with both FL and US (4). It is a more favourable option for patients with lower lumbar radicular pain accompanying coccydynia. In terms of efficacy, Sencan et al. compared GiB with caudal epidural steroid injection in the treatment of coccydynia and found that GiB was more effective [21]. Kaya et al. found that adding caudal epidural steroid injection to GiB did not contribute to the treatment of coccydynia [26].

Based on the available information, the GiB seems to be the most effective interventional procedure among those discussed above for coccydynia. The procedure can be performed using various approaches, including transcoccygeal and sacrococcygeal, and can also be guided by FL or US. Ghai et al. stated no complications in 15 patients who underwent GiB with US and reported US-guided intervention as a safe method. However, considering its close proximity to the rectum, we believe that FL-guided intervention is more reliable [27,28].

In 2021, two meta-analyses of coccydynia treatment were published by Andersen and Choudhary [6,19]. Andersen et al. reported that GiB was considered successful in 75% of 119 patients and Choudhary in >85% of 104 patients. Both authors reported that the change in pain score decreased by an average of 58%-60% between week 4 and week 12, and this rate was 37%-38% at week 24. Choudhary also notes a complication rate of 2.8% with GiB. Minor side effects such as vasovagal reaction with ganglion block, transient increase in pain, bradycardia, and hypotension have been reported [29,30]. Although rare, serious complications such as rectal perforation, bleeding, infection, bladder incontinence, sexual dysfunction, and nerve root injury may occur [31-34]. According to our results, in the GiB group, the NRS score improved by 60.22% at one month and 58.37% at three months compared to baseline.

The coccygeal nerves arise from the coccygeal plexus and are responsible for pain sensation in this region. After emerging from the sacral hiatus, they become superficial as they follow a mediolateral course through the coccygeal cornuas. This superficial course makes it easy to reach the nerve with US [16].

Although Gruber et al. said that the coccygeal nerves can be reached above the sacral cornu by US, sometimes the coccygeal nerves are located between the sacrococcygeal ligament and the medial wall of the sacral cornu at this level [19,35]. This can make it difficult to reach the nerve.

Domingo-Rufes et al. described a US-guided block of the coccygeal nerves at the level of the sacral cornu. They visualised the sacral hiatus and the sacrococcygeal ligament with US and inserted the needle into the sacral canal along the medial edge of the sacral cornu using an out-of-plane approach. However, there is a risk of caudal epidural puncture if the needle tip passes the S2 level [36].

Recently, Wu et al. published a paper in which they blocked the coccygeal nerves at the level of the coccygeal cornu under US guidance in a case of coccydynia. In this article, it is stated that the nerve trapped in the medial part of the coccygeal cornu can be visualised with the US probe and is safe in terms of the risk of epidural puncture as the injection site is outside the sacral hiatus. After two local anaesthetic injections two weeks apart, visual analogue scale (VAS) scores decreased by more than 50% [19].

Following Wu's case report, Can et al. published an article with 32 patients [20]. In this study, patients with coccydynia underwent conventional RFT neurotomy (90°C for 60 seconds) and steroid injection of the coccygeal nerves at the level of the coccygeal cornuas under US guidance. VAS and PARIS functional scores were followed for 12 weeks. There was a statistically significant improvement in both scales. According to our results, in the CnB group, there was an improvement of 49.35% in NRS scores at four weeks and 46.23% at 12 weeks. At 12 weeks, there was an improvement of 41.60% in the PARIS Functionality Scale. Can et al. showed a 57.77% improvement in VAS pain score at four weeks compared to baseline, and this was maintained at 12 weeks. The PARIS Functionality Scale showed a 50.74% improvement at 12 weeks compared to baseline. The efficacy of treatment achieved by Can et al. was better and longer lasting than our results. We attribute this to their application of conventional radiofrequency to the coccygeal nerves. The efficacy achieved by creating permanent nerve damage was still present at four weeks. However, there was an increase in our NRS scores at 12 weeks compared to four weeks. This suggests that the application of radiofrequency to the coccygeal nerves in the treatment provides longer and more successful results.

In terms of side effects, although GiB is usually associated with minor side effects, serious side effects such as rectal perforation, cauda equina syndrome, and neuritis have been reported [37]. While performing a GiB in one patient, the needle tip passed through a narrow coccygeal vertebral joint space and was forcefully advanced beyond the anterior line of the joint. The rectum was not perforated, but prophylactic antibiotic treatment was started. Needle control may be lost or the procedure may be traumatic due to excessive strain during needle insertion through an excessively angular or narrow coccygeal joint space. This can lead to increased pain after the procedure. One of our patients who had a difficult attempt in this way had an increase in pain for one week, and his complaints resolved with NSAII. 

Regarding adverse events associated with coccygeal nerve procedures, paraesthesias and increased pain were reported in three patients in the study by Can et al. In the CnB group in our study, two patients developed paraesthesia and one patient developed hyperalgesia at the injection site, but no treatment was required. In terms of adverse events, we had only minor adverse events like Can et al. We had two complications requiring treatment in the GiB group, but all adverse events resolved spontaneously without treatment in the CnB group.

The superiority of the CnB over the GiB was that it was performed under US guidance. The absence of radiation exposure is an advantage for both the practitioner and the patient. Although it is said that the GiB can also be performed with US, there is a serious risk of major complications. In addition, the superficial course of the coccygeal nerve means that it is sufficient to advance the needle tip about 1 cm during the procedure. This results in the patient experiencing less pain during the procedure. In our clinical practice, the CnB was easier to perform than the GiB.

Our study had several limitations. First,, we could not evaluate the effect of interventions on analgesic consumption. Second, we could not evaluate the effectiveness of treatments according to aetiologic perspectives. Third, we could not evaluate the effect of the interventions on the quality of life, mood, anxiety, and sleep quality.

## Conclusions

There are many invasive methods that can be used in patients with chronic coccydynia who do not respond to conservative treatment. However, nerve block at the level of the coccygeal cornu is a relatively new method. The present study is the first randomised controlled trial to assess the effectiveness of this new method. The results of this study do not provide evidence that GiB and CnB are superior to each other in terms of pain relief. The possible advantages of CnB include no radiation exposure, the site is far from the epidural space, no contrast is required, and it is easier to use because of the superficial course of the nerve. Further studies will increase our knowledge of the success of this method.
